# Cross-polarized photon-pair generation and bi-chromatically pumped optical parametric oscillation on a chip

**DOI:** 10.1038/ncomms9236

**Published:** 2015-09-14

**Authors:** Christian Reimer, Michael Kues, Lucia Caspani, Benjamin Wetzel, Piotr Roztocki, Matteo Clerici, Yoann Jestin, Marcello Ferrera, Marco Peccianti, Alessia Pasquazi, Brent E. Little, Sai T. Chu, David J. Moss, Roberto Morandotti

**Affiliations:** 1 Institut National de la Recherche Scientifique — Énergie Matériaux et Télécommunications, Université du Québec, 1650 Boulevard Lionel-Boulet, Varennes, Québec, Canada J3X 1S2; 2 Institute of Photonics and Quantum Sciences, Heriot-Watt University, Edinburgh EH14 4AS, UK; 3 Department of Physics and Astronomy, University of Sussex, Falmer, Brighton BN1 9RH, UK; 4 Xi'an Institute of Optics and Precision Mechanics of CAS, Xi'an 710119, China; 5 Department of Physics and Material Science, City University of Hong Kong, Tat Chee Avenue, Hong Kong, China; 6 School of Electrical and Computer Engineering, RMIT University, Melbourne, Victoria 3001, Australia; 7 Institute of Fundamental and Frontier Sciences, University of Electronic Science and Technology of China, Chengdu 610054, China

## Abstract

Nonlinear optical processes are one of the most important tools in modern optics with a broad spectrum of applications in, for example, frequency conversion, spectroscopy, signal processing and quantum optics. For practical and ultimately widespread implementation, on-chip devices compatible with electronic integrated circuit technology offer great advantages in terms of low cost, small footprint, high performance and low energy consumption. While many on-chip key components have been realized, to date polarization has not been fully exploited as a degree of freedom for integrated nonlinear devices. In particular, frequency conversion based on orthogonally polarized beams has not yet been demonstrated on chip. Here we show frequency mixing between orthogonal polarization modes in a compact integrated microring resonator and demonstrate a bi-chromatically pumped optical parametric oscillator. Operating the device above and below threshold, we directly generate orthogonally polarized beams, as well as photon pairs, respectively, that can find applications, for example, in optical communication and quantum optics.

## Abstract (web summary)

On-chip nonlinear optics devices find a number of applications in modern optics from spectroscopy to communications. Here, the authors increase the degrees of freedom for frequency mixing by demonstrating the nonlinear interaction of perpendicularly-polarized modes in an integrated microring resonator.

## Graphical abstract

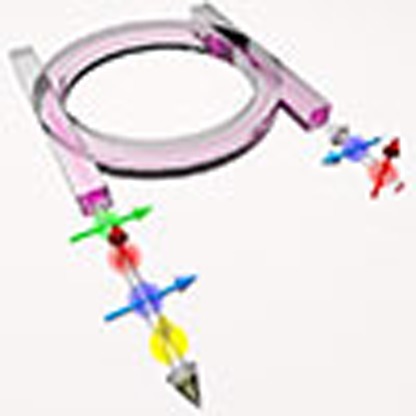

Following the ground-breaking demonstration of second-harmonic generation by Franken *et al*.^1^, nonlinear optical processes quickly rose to form the backbone of disciplines as important as spectroscopy^2^, signal processing^3^ and quantum optics^4^. With the goal of compact, more stable and scalable devices, the main platform for nonlinear optical architectures has rapidly evolved from bulk optics to fibre-based devices^5^, and has more recently progressed towards integrated photonics^6^,^7^. In particular, devices compatible with large-scale electronic chip complementary metal–oxide–semiconductor (CMOS) technology offer the potential for both mass production and low-cost commercial implementation^8^. Since second-order nonlinear materials are very challenging to integrate and are typically not CMOS compatible^8^, most integrated nonlinear devices rely on third-order processes. Various third-order nonlinear processes such as four-wave mixing (FWM), self- and cross-phase modulation, Raman and Brillouin scattering have been exploited on chip^9^ to achieve significant breakthroughs such as integrated broadband nonlinear parametric gain^10^, optical parametric oscillation^11^, frequency combs^12^, third-harmonic generation^13^, optical modulators^14^, all-optical routing^15^, mode-locked lasers^16^, photon pair sources^17^ and many others.

Most applications exploiting nonlinear processes in either bulk media or fibre-based devices have extensively relied on the electric field polarization as a fundamental degree of freedom to achieve novel nonlinear functionalities^5^. There are several different types of FWM, as well as spontaneous parametric downconversion, which can be categorized, for example, by the frequency and polarization of the interacting fields^5^,^18^,^19^. In general, the fields generated through parametric frequency conversion can either have identical (degenerate) or different (non-degenerate) frequencies and present different combinations of polarization, according to the following definitions: Type-0, the pump and generated fields are co-polarized; Type-I, the generated fields are co-polarized but different from the pump polarization; and Type-II, the generated fields have orthogonal polarizations. Table 1 summarizes the different types of FWM processes in terms of linear polarization for the different pump and generated fields (horizontal and vertical), as well as their efficiency assuming equal and perfect phase-matching conditions^20^. All these three polarization combinations can be achieved in second-order nonlinear media^21^, enabled by the crystal symmetry, and have been used, for example, to generate (entangled) photon pairs. In contrast, integrated third-order devices have not been able to exploit all of these degrees of freedom. While Type-II spontaneous FWM could in principle be achieved by using two orthogonally polarized pump fields^20^,^22^, the overlapping and dominant stimulated processes generally make spontaneous FWM experimentally undetectable^21^,^23^. Furthermore, to achieve efficient frequency conversion in a small footprint, integrated nonlinear processes are often enhanced through the use of cavities^16^,^24^ or photonic crystal waveguides^13^. These structures are typically designed for single polarization operation, as most integrated waveguides show a strong polarization-dependent dispersion and loss. Achieving efficient Type-II spontaneous FWM on a chip therefore requires structures that not only operate on two orthogonal polarizations with specific dispersion properties, but also provide nonlinear enhancement while suppressing competing stimulated processes.

Here we demonstrate cross-polarized spontaneous FWM in a novel bi-chromatically pumped integrated optical parametric oscillator (OPO). To achieve this, we introduce a method to suppress stimulated degenerate FWM while enhancing spontaneous non-degenerate FWM between two cross-polarized pumps inside a high-Q integrated nonlinear microring resonator. We show, on the one hand, that this novel kind of OPO emits high-purity orthogonally polarized photon pairs when operated below OPO threshold. On the other hand, it generates orthogonally polarized beams while running above the OPO threshold. The investigated device and the implementation of the cross-polarized FWM process can find various applications in quantum optics, as well as optical communications.

## Results

### Type-II SFWM

**Figure 1:**
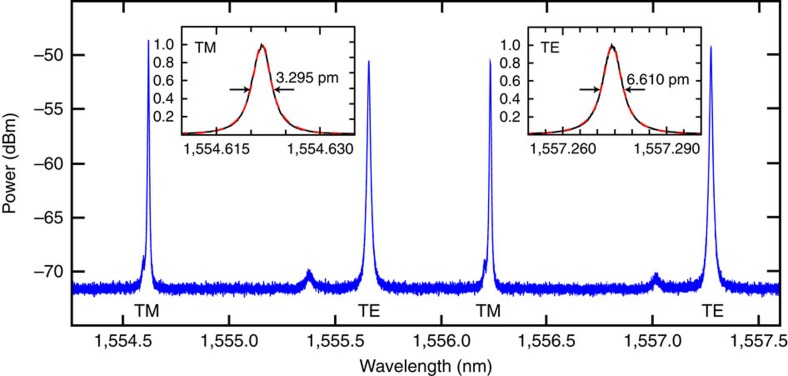
Microring resonator characteristics. Transmission spectrum measured with a high resolution Optical Spectrum Analyser (OSA), showing two TE and two TM resonances, with a relative frequency offset of 70 GHz. A very small amplitude, higher order mode excitation is also visible. However, these modes do not play any role in the FWM process due to the requirement of energy conservation. The insets show the TE and TM resonances in a linear scale (black) with a Lorentzian fit (red dashed).

**Figure 2:**
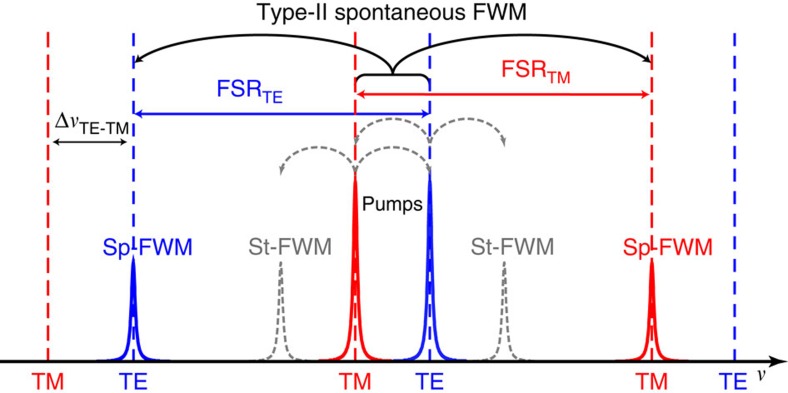
Schematic of the novel approach used to achieve Type-II spontaneous FWM on a chip. Stimulated FWM (St-FWM, dotted lines) is suppressed by an offset between the TE and TM resonances (dashed vertical lines), as the St-FWM gain does not overlap with any of the microring resonances. Correspondingly, Type-II spontaneous FWM (Sp-FWM, continuous line) is allowed and enhanced by the resonator.

Here we demonstrate Type-II spontaneous FWM in an integrated photonics platform, and achieve a novel kind of OPO—specifically a cross-polarized bi-chromatically pumped OPO. Our approach is based on a microring resonator fabricated in a CMOS-compatible high refractive index glass^8^ (Hydex, see Methods). The microring resonator operates on both fundamental transverse electric (TE) and transverse magnetic (TM) modes having similar yet slightly different dispersions. Cavity enhancement is provided by the high Q-factors of the TE and TM resonances that enable high parametric gain at low pump powers. The measured spectral response of the resonator is presented in Fig. 1. The insets show the measured TE and TM resonances (black) plotted in a linear scale with a Lorentzian fit (red dashed), revealing resonance bandwidths of 6.610 pm (820 MHz) and 3.295 pm (410 MHz), which result in measured Q-factors of 235,000 and 470,000, respectively. In our scheme, the suppression of stimulated FWM between the two pumps was obtained by generating a frequency offset of 70 GHz between the TE and TM resonances, while keeping the free spectral ranges (FSRs) of both modes almost identical (200.39 and 200.51 GHz, respectively), allowing Type-II spontaneous FWM to take place at targeted resonances, see Fig. 2 (for more details, see Methods). This offset is generated by the slightly different dispersions of the TE and TM modes, resulting in different effective resonator lengths and hence different resonant frequencies. Energy conservation dictates that the stimulated FWM bands have to be symmetric with respect to the two pump frequencies. Due to the frequency offset between the TE and TM resonances the spectral position of the stimulated FWM gain does not overlap with the ring resonances, thus suppressing this process inside the microring resonator. At the same time, the TE and TM mode dispersion has to be kept similar so that the difference in FSR between the two modes (120 MHz) is smaller than the bandwidth of the resonances, to achieve energy conservation for Type-II spontaneous FWM processes. Furthermore, the mismatch between the FSRs with respect to the resonator bandwidth has to be minimized to achieve high efficiency in Type-II FWM (see Methods). Finally, the required phase-matching condition can be achieved by operating in a slightly anomalous dispersion regime for both modes^5^.

### Below-threshold operation

**Figure 3:**
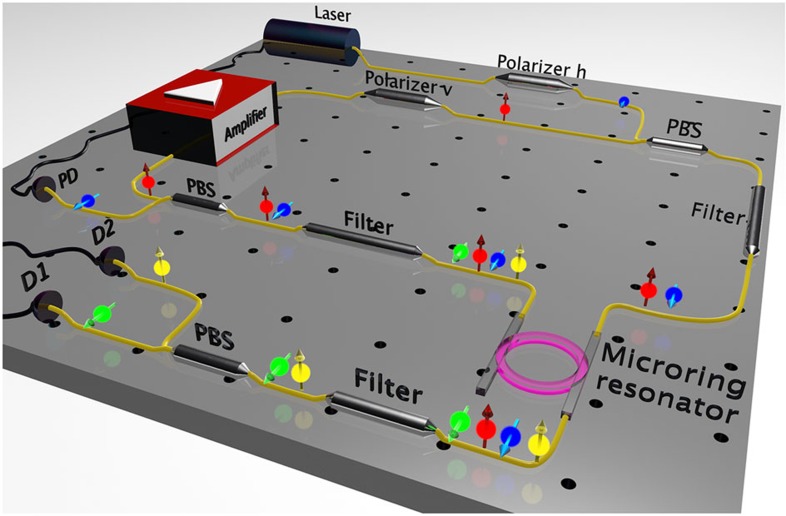
Experimental set-up of the hybrid self-locked and external pumping scheme. The TE polarization is pumped in a self-locked scheme^16^,^24^,^38^, while the TM pump field (a CW external fibre laser actively locked to the resonance) is added and extracted by two polarization beam splitters (PBSs) placed before and after the microring resonator. The amplified spontaneous emission of the amplifier is transmitted through the band-pass filter before the resonator, thus selecting the desired pump resonance. The output of the resonator is then fed back into the amplifier and acts as a seed to initiate lasing on the TE resonance. The photon pairs are extracted at the through port of the resonator and directed, after filtering out the pump fields, to detectors D1 and D2. The arrow on top of the amplifier represents the propagation direction of the light inside the cavity. The coloured spheres with arrows illustrate the frequency and polarization of the involved fields: red and blue are the TE and TM pumps, respectively, while yellow and green are the TE and TM daughter photons, respectively, generated through Type-II spontaneous FWM.

**Figure 4:**
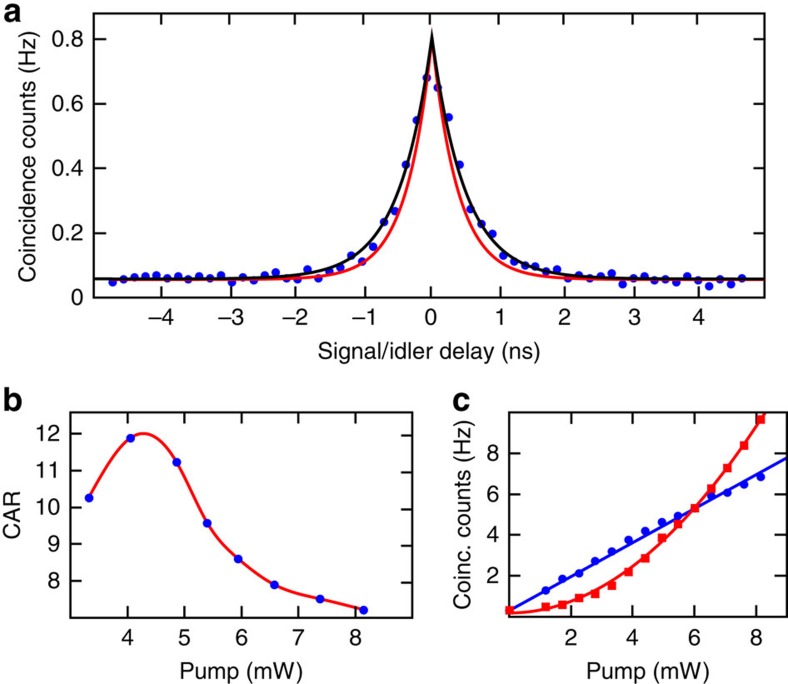
Photon pair source characterisation. (**a**) Measured photon coincidence peak, showing the raw measured coincidences (*C*) in Hz. The black curve corresponds to the optimum fit resulting in a measured photon bandwidth of 320 MHz, while the red curve corresponds to the fit with the expected photon bandwidth of 410 MHz. (**b**) CAR (coincidence-to-accidental ratio) as a function of balanced pump power (the line connecting the points is just for visual purposes), showing a CAR >10 for balanced pump powers between 3 and 5 mW. (**c**) Measured photon coincidence counts (sum of all coincidence counts measured within the FWHM of the coincidence peak) for balanced and unbalanced pump powers. In the unbalanced configuration (blue circles), the TE pump power is kept constant at 6 mW and the TM pump power is increased, showing a linear scaling behaviour. In the balanced configuration (red squares), TE and TM pump powers are identically increased, showing a clear quadratic scaling behaviour without any linear contribution.

When operated below the OPO threshold, our device directly generates orthogonally polarized photon pairs. To characterize them and confirm the nature of the underlying nonlinear process, we performed photon coincidence measurements (see Methods). The photon pairs generated in the TE and TM modes of the microring resonator were collected at the ring through port after appropriate filtering of both pump fields by means of a polarization-maintaining, high-isolation 200-GHz-wide notch filter (for example, by TeraXion Inc.). The device was pumped in a hybrid self-locked pump configuration (Fig. 3), where the TE pump laser (1,555.65 nm) was directly built around the resonator, while the TM resonance (1,556.24 nm) was pumped with an external laser (see Methods). The generated photons were then separated by a polarizing beam splitter and detected with single-photon detectors. We measured a clear coincidence peak (see Fig. 4a) with a coincidence-to-accidental ratio (CAR) of up to 12 without any background subtraction (see Fig. 4b). As photon pairs can only be generated via spontaneous nonlinear processes, the measured photon coincidences give a strong indication that the photon pairs are generated through Type-II spontaneous FWM, with stimulated processes being successfully suppressed. The power-scaling behaviour provides further insight into the process associated with the generation of the photon pairs. Only when one photon from each pump field is used to create two daughter photons does it become possible to directly generate orthogonally polarized photon pairs^20^. Therefore, the coincidence counts (*C*) are expected to scale with the product of both pump powers^5^ (*C*∝*P*_TE_ × *P*_TM_). If the power of one pump field is kept constant, and the power of the second one is increased, a linear scaling behaviour is predicted for type-II spontaneous FWM, whereas if the power of both pump fields is simultaneously increased (with constant power ratio), a quadratic scaling is expected. As shown in Fig. 4c, no coincidences (within the noise) were measured when the ring was not pumped or pumped with the TE field alone, where the non-zero counts are due to the dark counts of the detector. A clear linear scaling behaviour is visible with increasing TM pump power and constant TE power, while a quadratic (without linear contribution) scaling is observed with increasing balanced pump powers. The presence of Raman scattering can be neglected, as the signal and idler frequencies do not overlap significantly with the Raman gain spectrum. This is also experimentally confirmed by the absence of any linear contribution to the power scaling^20^ arising from Raman scattering.

### Heralded photon source

**Figure 5:**
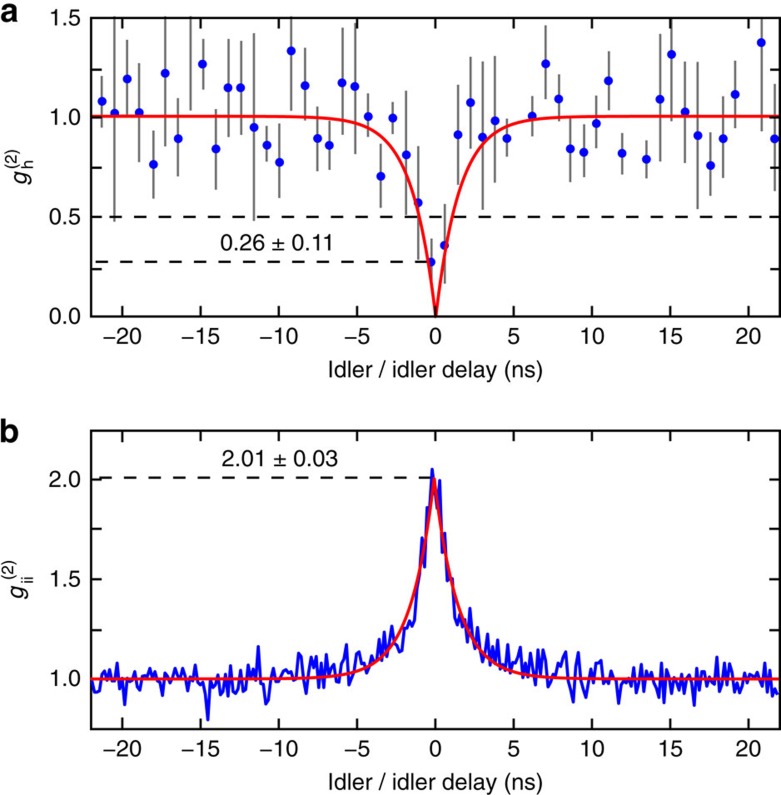
Heralded and idler–idler autocorrelation measurement. (**a**) Measured heralded autocorrelation, showing a clear dip at zero delay below the limit for classical correlations (equal to 0.5), confirming the quantum nature and single-photon operation of the source. Ten bins were averaged for each point, displayed together with the statistical error (standard deviation of the 10 bin distribution). (**b**) Measured idler–idler autocorrelation, showing a clear peak with a maximum at 2.01±0.03, confirming the single-mode operation and high purity of the source.

**Figure 6:**
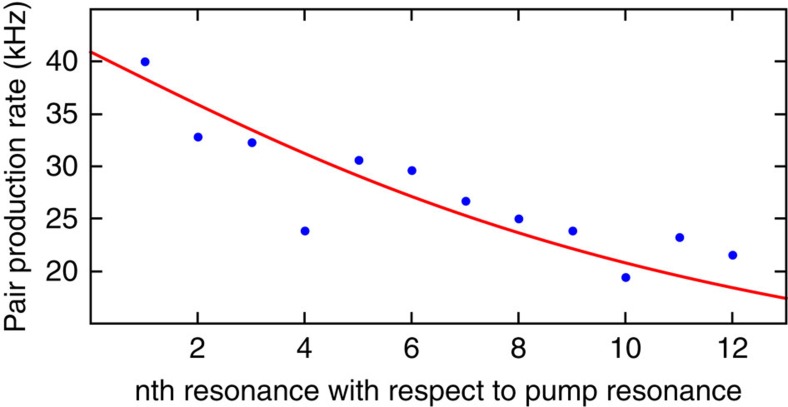
Pair production rate at different resonances. Measured pair production rate (blue circles) associated to the Type-II process at different resonator lines symmetrically located with respect to the pumps at 5 mW balanced pump power, showing good agreement with the approximated predicted curve (red line), see Methods for details.

In addition to revealing the nature of the nonlinear process, photon pairs are of interest for several applications, such as quantum communications^25^. Orthogonally polarized photon pair generation has recently been demonstrated in non-CMOS compatible second-order nonlinear integrated Bragg reflection waveguides using Type-II parametric downconversion^26^. In third-order nonlinear media, orthogonally polarized photon pairs have been generated in specifically designed non-resonant microstructured fibres pumped at 800 nm (ref. 27). On a third-order nonlinear chip, the superposition of two Type-0 nonlinear waveguide sources has been achieved, where two straight waveguides were connected by a polarization rotation segment to generate photon pairs, which are either both TE or TM polarized^28^. In stark contrast, the source presented here directly generates orthogonally polarized photon pairs (one TE, one TM) on chip with a single third-order process. Due to the difference in linewidth between the TE and TM resonances, the spectral bandwidth of the emitted photons is determined by the narrower resonance. From the coincidence measurement, shown in Fig. 4a, we extract a measured photon bandwidth of 320 MHz (black line), which is in good agreement with the resonator bandwidth of 410 MHz (red line), where the difference can be explained by the timing jitter of the detectors and electronics, resulting in a small temporal broadening of the measured peak. It is worth noting that the narrow bandwidth, required for several quantum applications^29^, is intrinsically achieved inside the resonator^24^ and cannot be directly realised in non-resonant waveguides or fibre-based architectures. The measured CAR of up to 12 is limited by loss, dark counts and the quantum efficiency of the detectors (see Methods), as well as by the photons generated through Type-0 SFWM of the individual pumps, that is, issues which can in the future be addressed by optimized dispersion control. However, we note that a CAR >10 already suggests the possibility for an immediate implementation of the source for quantum cryptography applications, see, for example, ref. 25. We measure a coincidence rate of ∼4 Hz at 5 mW balanced pump power at the input of the chip (5 mW is the highest achievable pump power featured by a CAR >10). Considering all losses of the detection system (8.5 dB for both signal and idler) as well as the quantum efficiency of the detectors (5 and 10%), this corresponds to a pair production rate (PPR) of 40 kHz and a pair production probability of 1.48 × 10^−12^, accounting for the 1.6 dB coupling loss of the pump into the chip. The measured coincidence rate can be further increased to approach the production rate by using better detectors and implementing low-loss filtering on chip. To further characterize the performance of our device as a single-photon source, we measured the heralded autocorrelation function *g*_h_^(2)^, as well as the idler–idler autocorrelation function to estimate the purity of the state (see Methods). A clear dip of *g*_h_^(2)^(0)≃0.26±0.11<0.50 was recorded (see Fig. 5a), showing that the source operates in a non-classical single-photon regime^29^,^30^, while the idler–idler autocorrelation (see Fig. 5b) shows a clear peak with a maximum of 2.01±0.03, resulting in *N*=0.99±0.03≈1 effective modes, underlining the high purity of the source. Finally, the production of cross-polarized photon pairs is not limited to only the adjacent resonances, but the generation of multiplexed cross-polarized photon pairs is also possible. Indeed, we measured cross-polarized photon pairs over 12 resonance couples, limited by the available filters, each with PPRs >20 kHz at 5 mW balanced pump power (see Fig. 6 and Methods). All these characteristics highlight the potential of our device for quantum optical applications.

### Above-threshold operation

**Figure 7:**
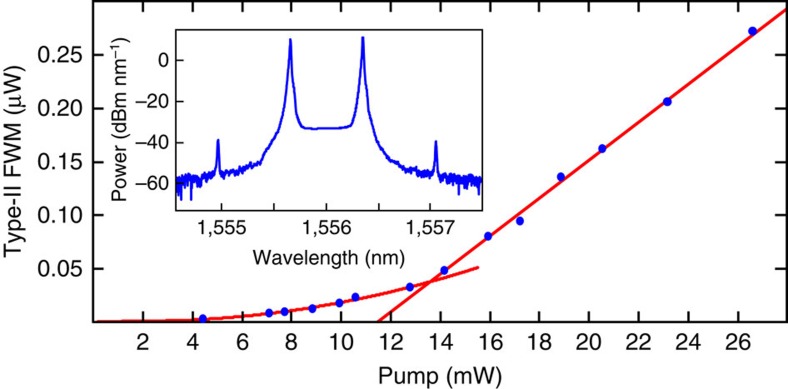
Cross-polarized optical parametric oscillation. Power scaling in a high-Q ring resonator with balanced pumps, initially showing a quadratic followed by a linear trend above OPO threshold at 14 mW. The inset presents the OPO spectrum measured with an optical spectrum analyser at 20 mW pump power.

The same pumping scheme and Type-II FWM process can, in principle, lead to above-threshold OPO. However OPO operation could not be reached at the available pump powers (up to 26 mW) with the resonator used in the experiments mentioned above. We therefore resorted to a second ring with higher Q-factors of 750,000 and 1,100,000 for the TE and TM modes, respectively, which was pigtailed to single-mode, non polarization-maintaining fibres, thus preventing us to use this device for the single-photon experiments. Instead of separating the beams by polarization, we detected all outputs (pump and generated fields) using an optical spectrum analyser. With a balanced pump power, a quadratic power-scaling behaviour was measured below threshold (Fig. 7), as also seen in Fig. 4c for the low-Q ring. At the OPO threshold, which was reached at 14 mW balanced pump power (see OPO spectrum in the inset of Fig. 7), the power scaling changed from quadratic to linear, confirming the transition from spontaneous emission to OPO (ref. 11). This device is a novel type of bi-chromatically pumped OPO operating on two orthogonally polarized beams.

## Discussion

We achieve Type-II spontaneous FWM in an integrated platform, thus providing more access to polarization as a degree of freedom for integrated third-order spontaneous nonlinear interactions. Using this process, we demonstrate a novel bi-chromatically pumped OPO, which below threshold directly generates orthogonally polarized photon pairs on a CMOS-compatible chip. The measured photon bandwidth, CAR and high purity single-mode operation in the non-classical single-photon regime underline the utility of the photon pair source for quantum applications. For example, on-chip wavelength photon routing was recently achieved using electronically controlled spectral filters^31^. With our cross-polarized source, polarization photon routing and quantum operations can be achieved by using the source in combination with passive and easy-to-implement polarization elements such as polarizing beam splitters^32^,^33^ and waveplates^34^. Furthermore, since with our scheme two different FWM processes become accessible on the same chip, our device opens up the possibility of using, for example, Type-0 and Type-II FWM simultaneously to generate complex quantum optical states (for example, multi-entangled states) on a compact platform.

Above threshold, the novel OPO directly generates orthogonally polarized beams, which can open up a new route for the generation of polarization-squeezed light or find applications in polarization-multiplexed and coherent communications. Finally, as Type-II FWM is a fundamental nonlinear process, besides the realization of a cross-polarized OPO, different devices and geometries can be realized that lead to further applications ranging from entanglement generation to parametric amplifiers and all-optical signal processing.

## Methods

### Device fabrication

The microring resonators are fabricated using UV photolithography and reactive ion etching in a CMOS-compatible high refractive index silica glass deposited by chemical vapour deposition without the need for high temperature annealing. Hydex is featured by very low linear (<0.06 dB cm^−1^)^8^ and negligible nonlinear optical losses (no nonlinear losses measured up to 25 GW cm^−2^)^8^, and a high effective nonlinearity (*γ*=*ωn*_2_/(*c*_0_*A*_eff_)≈233 W^−1^ km^−1^)^8^. The etched waveguide cross-section is almost square (1.5 × 1.45 μm), in turn enabling the desired slightly different dispersions in the TE and TM modes, which are low and anomalous at 1,550 nm for both polarizations (zero dispersion wavelengths at 1,560 nm and 1,590 nm, respectively)^8^. The microring resonators are vertically coupled to two bus waveguides, forming a four-port configuration. The resonator used for the single-photon measurements exhibits 200.39 GHz and 200.51 GHz FSR with an offset of 70 GHz, as well as Q-factors of 235,000 and 470,000 (820 MHz and 410 MHz bandwidth) for the TE and TM modes, respectively. The input and output bus waveguides are featured with mode converters and are pigtailed to polarization-maintaining fibres, resulting in coupling losses of <1.6 dB per facet. The resonator used for the above-threshold OPO measurement exhibits 200.54 and 200.76 GHz FSR with an offset of 85 GHz, as well as Q-factors of 750,000 and 1,100,000 for the TE and TM modes, respectively. The input and output bus waveguides are pigtailed to single-mode fibres (non polarization maintaining), resulting in coupling losses of <1.5 dB per facet.

### Type-II FWM and suppression of stimulated FWM

In addition to phase matching, the energy has to be preserved in all nonlinear processes. For the two pumps involved in the Type-II FWM process, the total input pump energy is given by the sum of both pump photon energies


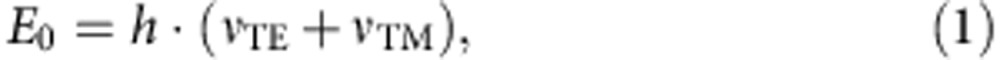


*h* being the Planck's constant, and *ν*_TE_ and *ν*_TM_ the central frequencies of the two pump resonances.

As the pump resonances have a specific linewidth, and assuming a Lorentzian resonance (as in our case, see the spectrum in Fig. 1), the energy bandwidth is given by the convolution of both pump lines, which for two Lorentzian curves is also given by a Lorentzian


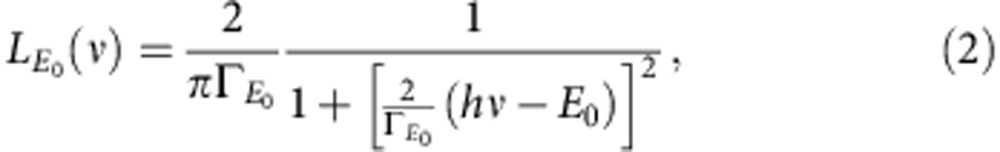


featured by a full width at half maximum (FWHM) of:


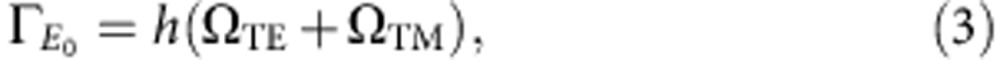


with Ω_TE,TM_ being the frequency FWHM of the individual resonances. The same can be estimated for the nth adjacent resonances, where the signal and idler photons will be generated. The total output energy curve will be again a Lorentzian


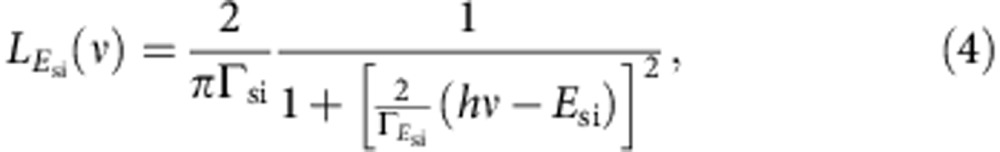


centred at


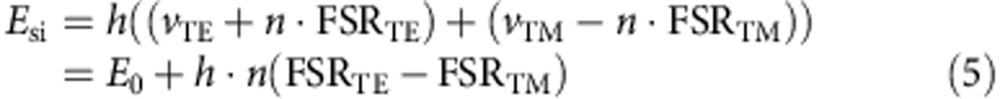


with a FWHM


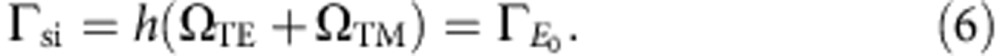


To achieve spontaneous FWM, energy conservation must be fulfilled, that is, the energy curves 
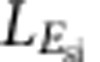
 and 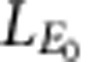 have to overlap. The overlap integral for the two Lorentzian curves (that is, the area of the overlapping region of 
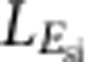
 and 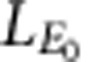) is given by:


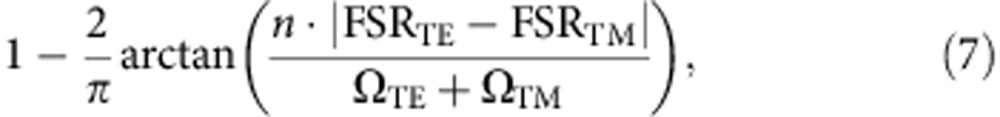


which becomes 1 for equal FSRs in TE and TM, and decreases when the mismatch between FSRs with respect to the FWHM of the resonances increases.

In addition to the overlap, the resonator linewidth and Q-factor play an important role in the definition of the spontaneous FWM process efficiency. When the Q-factors of the resonances are the same, the FWM efficiency is expected to scale with the Q-factor to the power of 4 (ref. 35). In the case where the TE and TM resonances have different bandwidths and Q-Factors, the FWM process is expected to scale as 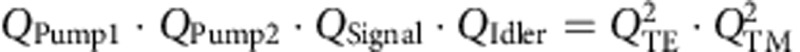 for the Type-II FWM process.

Using the measured values (see main text), the PPR for different resonances with respect to the offset can be approximated with:


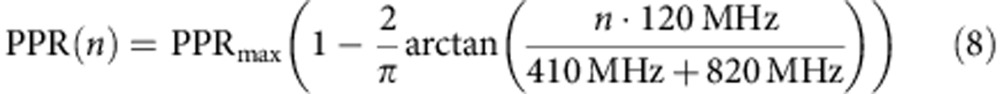


Note that this approximation does not include higher order dispersion and assumes a flat FWM gain spectrum. Even with these assumptions, the fit in Fig. 6 shows good agreement to the measured data, resulting in a PPR_max_=40.92±1.33 kHz extracted from the fit.

Stimulated FWM can be fully suppressed by designing the ring resonator in such a way that no ring resonance overlaps with the stimulated FWM bandwidth. With two pump frequencies *ν*_TE_ and *ν*_TM_ separated by Δ*ν*_TE–TM_ the frequencies for stimulated FWM are:


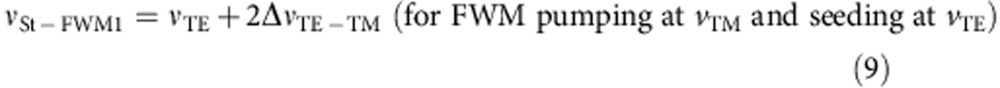



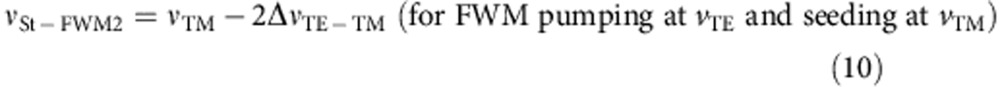


with a linewidth of


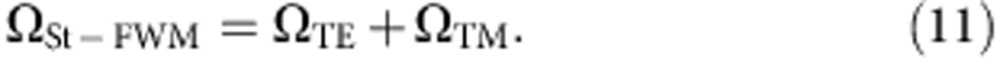


The stimulated FWM bandwidths do not overlap, and thus stimulated FWM is suppressed, under the following assumptions:


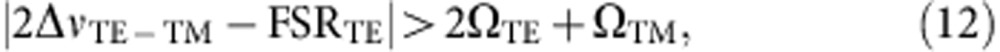



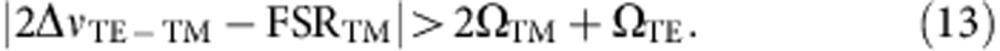


The FSRs and Δ*ν*_TE−TM_ depend on the waveguide dispersion, while the FWHM depends on linear and bending losses together with the resonator coupling. Therefore, the above stated equations can be used to design the ring resonator to simultaneously achieve both cavity enhancement of the spontaneous Type-II FWM and complete suppression of the stimulated FWM between the two pumps.

For example, for the first (lower-Q) microring resonator, equations (12) and (13) are satisfied since:


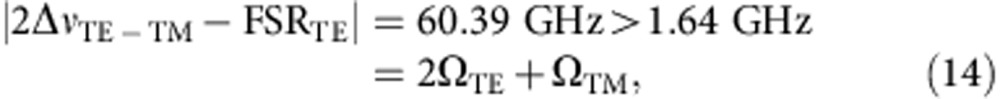



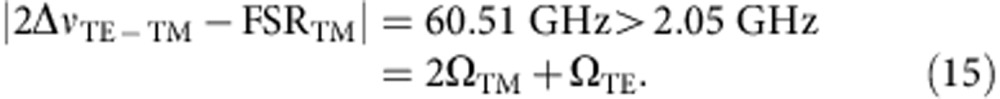


The same also holds for the second (higher Q) microring resonator.

### Single-photon measurements

The coincidence measurements were done using two single-photon detectors (idQuantique id210), one set to the free-running mode with 5% quantum efficiency leading to 1.6 kHz dark-count rates, while the second detector is triggered by the first and operated at 10% quantum efficiency, resulting in 0.3 Hz dark coincidence counts. Time tags from both detectors were collected using a time-to-digital converter with 81 ps timing resolution (idQuantique id800). To realistically assess the properties of our device, unless explicitly stated, all measurements were performed using raw data without background subtraction or correction for losses, detection efficiency or dark counts. For the heralded *g*_h_^(2)^ measurement, the photons were separated using a polarizing beam splitter, followed by a second 50:50 beam splitter in the idler arm. A third single-photon detector (idQuantique id201) was used, also triggered by the first detector measuring the signal photon. The heralded autocorrelation function *g*_h_^(2)^ can be directly extracted from the time tags using the relation^29^:


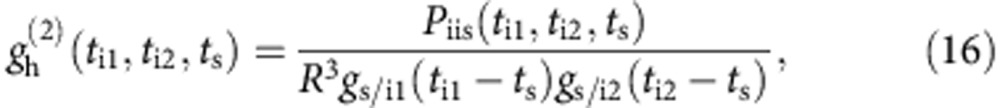


where *t*_i1(i2)_ are the detection times of the idler photon at the first (second) output port of the beam splitter, respectively, *t*_s_ is the detection time of the heralding signal photon, *g*_s/i1,2_ are the normalized Glauber cross-correlation functions, *P*_iis_*(t*_i1_*,t*_i2_*,t*_s_) is the triple coincidence rate and *R* is the PPR. To quantify the noise, 10 bins are averaged and the relative standard deviation is displayed in the error bars in Fig. 5a. It is important to note that the method described above to measure the heralded autocorrelation is not valid for all experimental set-ups^36^. It is for instance required that the photon coherence time is larger than the detection time-bin, the timing jitter and the heralding time window^29^,^36^, all necessary constraints which are fulfilled in our experiment.

For a perfect heralded photon source, it is expected that only one idler photon is present if a signal photon is detected, which results in a dip approaching zero in the conditional coincidence measurement (*g*_h_^(2)^(0)→0). In real systems, where we need to account for the possibility of generating multiple photon pairs, as well as for both losses and dark count detection, the visibility is reduced. However, a dip in the conditional coincidence function <0.5 (not corrected for any losses or low detection efficiencies) is sufficient to prove the quantum nature of a heralded photon source^30^. The dominant source of error originates from the fluctuations in the triple coincidence measurement, which are often caused by detector dark counts. Despite the fact that this measurement lasted 3 weeks, the low number of observed triple coincidences resulted in a high relative error.

The idler–idler autocorrelation function, *g*_ii_^(2)^, where the signal photon is not detected, can instead be used to reveal the purity of the state and the number of effective modes. After a photon pair is generated, there is a certain probability to stimulate the emission of a new pair. This results in an autocorrelation peak with a maximum related to the number of effective modes (*N*) through the relation *g*_ii_^(2)^(0)=1+1/*N*. A pure state is thus characterized by *g*_ii_^(2)^(0)=2, corresponding to a single mode^37^.

### Hybrid pumping scheme

The external pumping of high-Q microring resonators with a single pump laser usually requires thermal locking to follow the frequency shift of the resonances induced by cavity heating. Using two external CW lasers to simultaneously pump two resonances of the same device adds a significant degree of complexity leading to a very unstable operation. For this reason, we used a hybrid self-locked pumping approach, where the laser pumping the TE mode was directly built around the resonator, thus eliminating the need for active stabilization^16^,^24^,^38^. The microring resonator was embedded inside an external cavity that includes a fibre amplifier and a wavelength filter (nested cavity design, see Fig. 3). The amplified spontaneous emission of the fibre amplifier was transmitted through a band-pass filter (100 GHz) centred at the desired TE ring resonance and was then coupled into the chip. Light coupled out of the drop port of the ring resonator was fed back to the amplifier, thereby closing the external pump cavity and promoting lasing on the TE mode^16^,^24^,^38^. To allow self-locked lasing only on the TE polarization, while pumping the TM mode with an external laser (actively locked to the resonance using a feedback loop), polarizing beam couplers were placed before and after the ring resonator. This hybrid approach using one self-locked and one external pump permits pumping on both resonances in a very stable configuration and provides precise control over the individual pump powers.

## Additional information

**How to cite this article:** Reimer, C. *et al*. Cross-polarized photon-pair generation and bi-chromatically pumped optical parametric oscillation on a chip. *Nat. Commun.* 6:8236 doi: 10.1038/ncomms9236 (2015).

## Figures and Tables

**Table 1:** Different types of FWM processes in terms of the polarization of the interacting fields.

	P1	P2	S	I	Efficiency^20^
	H	H	H	H	
Type-0					∝(*γ* × *L*)^2^ *P*_1_ × *P*_2_
	V	V	V	V	
	H	H	V	V	
Type-I					∝(*γ* × *L*/3)^2^ *P*_1_ × *P*_2_
	V	V	H	H	
	H	V	H	V	
	H	V	V	H	
Type-II					∝(*γ* × *L*/3)^2^ *P*_1_ × *P*_2_
	V	H	H	V	
	V	H	V	H	

FWM, four-wave mixing.

List of different FWM processes and their relative efficiencies (from Lin *et al*.^20^) in terms of pump powers (P1 and P2), propagation length (*L*) and nonlinear parameter (*γ*). P1, P2, *S* and *I* represent the first pump, second pump, signal and idler photon, respectively. H denotes horizontal and V vertical polarization.
